# Supplementary material for: The DNA methylome of human sperm is distinct from blood with little evidence for tissue-consistent obesity associations
Source: PLoS Genet. 2020 Oct 13;16(10):e1009035. doi: 10.1371/journal.pgen.1009035 (PMC7584170; doi:10.1371/journal.pgen.1009035)
Supplement: S1 Text — (DOCX) [file pgen.1009035.s001.docx]

**S1 Text. Replication.**

The majority of DNA methylation differences observed between whole blood and sperm replicated in the lean replication (n = 21 lean males) and obesity groups (n = 22 obese and overweight males) across the 692,219 probes that survived quality control in these groups: 288,062 of significant sites that were also present in the replication groups showed significant differences between blood and sperm in the replication group (65%; P < 9 × 10^-8^, paired t-test), and 306,023 sites (69%) in the obesity group. The effect sizes at the 441,764 significant probes from discovery, which were also present in the replication groups, were highly correlated with those observed in the replication groups (lean group: *r* = 98%, *P* < 1.0 × 10^-50^; obese group: *r* = 0.99, *P* < 1.0 × 10^-50^; **S4** **Fig, S3 Table**).

Correlations between whole blood and sperm DNA methylation were replicated in the two replication groups. 1,250 of the 1,513 significantly correlated sites had also passed quality control in the replication groups and 455 (36%) of these were significantly correlated in the lean replication group (*P* < 9 × 10^-8^, Pearson’s product moment correlation), 502 (40%) in the obesity group (**S12 Table**). Given the reduced power to detect significant correlation in these two groups of reduced size, we further characterized sites showing very little evidence of correlation in the replication of groups (*r* < 0.3 in both groups). These 173 sites (14%) are all driven by sets of outliers in the discovery cohort resulting in bi- or tri-modal distribution in the discovery sample, that were not present in the replication groups (examples shown in **S6** **Fig**). The majority (127 sites; 73%) were characterized by a bimodal distribution with a single outlier in the discovery.
