# Supplementary material for: The DNA methylome of human sperm is distinct from blood with little evidence for tissue-consistent obesity associations
Source: PLoS Genet. 2020 Oct 13;16(10):e1009035. doi: 10.1371/journal.pgen.1009035 (PMC7584170; doi:10.1371/journal.pgen.1009035)
Supplement: S1 Table — Sites showing > 80% median DNA methylation were classified as “high”, sites with median beta < 20% methylation as “low”. Enrichments of each region amongst “high” and “low” methylation sites were calculated against the annotation of intermediately methylated sites (20–80% median DNA methylation) using a Fisher’s exact test. OR = odds ratio. (DOCX) [file pgen.1009035.s002.docx]

| CpG region | DNA methylation | P | OR |
| --- | --- | --- | --- |
| Island | High | 3.90E-15 | 0.85 |
| Island | Low | < 1.00E-50 | 35.60 |
| Shore | High | < 1.00E-50 | 0.27 |
| Shore | Low | < 1.00E-50 | 1.95 |
| Shelf | High | 0.11 | 1.02 |
| Shelf | Low | < 1.00E-50 | 0.11 |
| Open sea | High | < 1.00E-50 | 2.19 |
| Open sea | Low | < 1.00E-50 | 0.07 |

**S1 Table. Enrichments of CpG region annotations across sites showing extreme methylation values in sperm.** Sites showing > 80% median DNA methylation were classified as “high”, sites with median beta < 20% methylation as “low”. Enrichments of each region amongst “high” and “low” methylation sites were calculated against the annotation of intermediately methylated sites (20-80% median DNA methylation) using a Fisher’s exact test.

*OR = odds ratio*
