# Supplementary material for: The DNA methylome of human sperm is distinct from blood with little evidence for tissue-consistent obesity associations
Source: PLoS Genet. 2020 Oct 13;16(10):e1009035. doi: 10.1371/journal.pgen.1009035 (PMC7584170; doi:10.1371/journal.pgen.1009035)
Supplement: S2 Table — Sites showing > 80% median DNA methylation were classified as “high”, sites with < 20% methylation as “low”. Enrichments of each region amongst “high” and “low” methylation sites were calculated against the annotation of intermediately methylated sites (20–80% median DNA methylation) using a Fisher’s exact test. OR = odds ratio. (DOCX) [file pgen.1009035.s003.docx]

| Region | DNA methylation | P | OR |
| --- | --- | --- | --- |
| TSS1500 | High | < 1.00E-50 | 0.41 |
| TSS1500 | Low | < 1.00E-50 | 2.12 |
| TSS200 | High | < 1.00E-50 | 0.66 |
| TSS200 | Low | < 1.00E-50 | 6.59 |
| 5'UTR | High | 2.21E-16 | 0.91 |
| 5'UTR | Low | < 1.00E-50 | 1.78 |
| Body | High | < 1.00E-50 | 1.59 |
| Body | Low | < 1.00E-50 | 0.40 |
| 1st exon | High | < 1.00E-50 | 0.67 |
| 1st exon | Low | < 1.00E-50 | 6.17 |
| Exon boundary | High | 5.39E-46 | 1.62 |
| Exon boundary | Low | < 1.00E-50 | 0.22 |
| 3'UTR | High | 3.81E-10 | 1.13 |
| 3'UTR | Low | < 1.00E-50 | 0.36 |
| Not annotated | High | < 1.00E-50 | 0.87 |
| Not annotated | Low | < 1.00E-50 | 0.31 |

**S2 Table. Enrichments of genomic region annotations across sites showing extreme methylation values in sperm.** Sites showing > 80% median DNA methylation were classified as “high”, sites with < 20% methylation as “low”. Enrichments of each region amongst “high” and “low” methylation sites were calculated against the annotation of intermediately methylated sites (20-80% median DNA methylation) using a Fisher’s exact test.

*OR = odds ratio*
