# Supplementary material for: The DNA methylome of human sperm is distinct from blood with little evidence for tissue-consistent obesity associations
Source: PLoS Genet. 2020 Oct 13;16(10):e1009035. doi: 10.1371/journal.pgen.1009035 (PMC7584170; doi:10.1371/journal.pgen.1009035)
Supplement: S4 Table — Reference ranges are derived from the UCLH Clinical Biochemistry Test Information sheet available from [70]. The reference range for HOMA-IR is derived from [71]. SD = Standard Deviation, IQR = interquartile range, BMI = Body Mass Index, SBP = Systolic Blood Pressure, DBP = Diastolic Blood Pressure, HOMA-IR = Homeostatic Model Assessment of Insulin Resistance, CRP = C-Reactive Protein, HDL = High Density Lipoprotein, LDL = Low Density Lipoprotein. (DOCX) [file pgen.1009035.s005.docx]

|  | Discovery group | Lean replication group | Obesity/ overweight  group | P  (difference between groups) | P  (discovery vs replication) | P (discovery vs obesity) | P  (replication vs obesity) |
| --- | --- | --- | --- | --- | --- | --- | --- |
| Age (years). Mean (SD) | 36.3 (5.2) | 34.1 (4.6) | 35.1 (4.1) | 0.192 |  |  |  |
| BMI (kg/m2). Mean (SD) | 23.4 (4.6) | 22.3 (1.1) | 29.1 (3.2) | <0.001 | 0.060 | <0.001 | <0.001 |
| Waist circumference (cm). Mean (SD) | 84.4 (4.8) | 82.4 (6.4) | 99.4 (8.7) | <0.001 | 0.436 | <0.001 | <0.001 |
| SPB (mmHg), average of two measurements. Mean (SD) | 119 (11) | 121 (10) | 126 (9) | 0.052 |  |  |  |
| DPB (mmHg), average of two measurements. Mean (SD) | 77 (8) | 78 (6) | 81 (8) | 0.050 |  |  |  |
| Total cholesterol (mmol/L). Mean (SD) | 4.7 (0.7) | 4.9 (0.9) | 4.9 (1) | 0.614 |  |  |  |
| HDL cholesterol (mmol/L). Mean (SD) | 1.6 (0.3) | 1.5 (0.3) | 1.4 (0.3) | 0.060 |  |  |  |
| LDL cholesterol (mmol/L). Mean (SD) | 2.7 (0.7) | 2.9 (0.8) | 2.9 (0.9) | 0.330 |  |  |  |
| Fasting glucose (mmol/L). Median (IQR) | 4.8 (0.5) | 4.6 (0.4) | 4.7 (0.6) | 0.018 | 0.003 | 0.088 | 0.105 |
| Fasting insulin (mIU/L). Median (IQR) | 5.3 (3.4) | 5.1 (3.0) | 8.9 (7.2) | 0.002 | 0.309 | <0.001 | 0.004 |
| HOMA-IR. Median (IQR) | 1.2 (0.8) | 1.1 (0.6) | 1.9 (1.4) | <0.001 | 0.285 | <0.001 | 0.005 |
| HOMA2-IR. Median (IQR) | 1.1 (0.5) | 0.6 (0.4) | 1.1 (0.9) | 0.014 | 0.048 | 0.414 | 0.003 |
| CRP (mg/L). Median (IQR) | 0.6 (0.3) | 0.6 (0.1) | 1 (1.8) | <0.001 | 0.105 | 0.001 | <0.001 |
| Triglycerides (mmol/L). Median (IQR) | 0.9 (0.5) | 0.9 (0.7) | 1.2 (0.6) | 0.282 | 0.335 | 0.056 | 0.157 |

**S4 Table. Phenotype characteristics of participants included in the discovery, replication and obesity groups.** Reference ranges are derived from the UCLH Clinical Biochemistry Test Information sheet available from [1]. The reference range for HOMA-IR is derived from [2].

*SD = Standard Deviation, IQR = interquartile range, BMI = Body Mass Index, SBP = Systolic Blood Pressure, DBP = Diastolic Blood Pressure, HOMA-IR = Homeostatic Model Assessment of Insulin Resistance, CRP = C-Reactive Protein, HDL = High Density Lipoprotein, LDL = Low Density Lipoprotein*
