# Supplementary material for: The DNA methylome of human sperm is distinct from blood with little evidence for tissue-consistent obesity associations
Source: PLoS Genet. 2020 Oct 13;16(10):e1009035. doi: 10.1371/journal.pgen.1009035 (PMC7584170; doi:10.1371/journal.pgen.1009035)
Supplement: S6 Table — Using a paired t-test the DNA methylation difference between the median methylation in blood and sperm was calculated for each region. The DNA methylation difference is shown with respect to blood (a positive value indicating higher average DNA methylation in sperm). (DOCX) [file pgen.1009035.s007.docx]

| Region | Probes | P | DNA methylation difference (%) |
| --- | --- | --- | --- |
| TSS1500 | 103,486 | < 1.00E-50 | -11 |
| TSS200 | 64,958 | < 1.00E-50 | -2 |
| 5'UTR | 92,296 | 3.61E-32 | 9 |
| Body | 297,434 | < 1.00E-50 | 2 |
| 1^st^ exon | 38,767 | < 1.00E-50 | -2 |
| Exon boundary | 6,462 | 2.80E-22 | 2 |
| 3'UTR | 20,248 | 3.81E-05 | -1 |
| Not annotated | 191,155 | < 1.00E-50 | 2 |

**S6 Table. Blood and sperm DNA methylation difference by genomic region.** Using a paired t-test the DNA methylation difference between the median methylation in blood and sperm was calculated for each region. The DNA methylation difference is shown with respect to blood (a positive value indicating higher average DNA methylation in sperm).
