# Supplementary material for: The DNA methylome of human sperm is distinct from blood with little evidence for tissue-consistent obesity associations
Source: PLoS Genet. 2020 Oct 13;16(10):e1009035. doi: 10.1371/journal.pgen.1009035 (PMC7584170; doi:10.1371/journal.pgen.1009035)
Supplement: S7 Table — The corresponding accession numbers are provided in S8 Table. (DOCX) [file pgen.1009035.s008.docx]

| Tissue | Number of samples |
| --- | --- |
| Adipose | 42 |
| Blood | 2317 |
| Brain | 868 |
| Buccal | 214 |
| Cartilage | 60 |
| Chorion | 3 |
| Colon | 170 |
| Epithelial | 183 |
| Fibroblast | 54 |
| Intestines | 1 |
| Kidney | 45 |
| Liver | 90 |
| Lung | 103 |
| Lymph node | 24 |
| Mucosa | 95 |
| Muscle | 17 |
| Neuron | 71 |
| Neutrophils | 69 |
| Pancreas | 112 |
| Rectum | 13 |
| Saliva | 146 |
| Skin | 38 |
| T cells | 136 |
| Unsorted cell lines | 9 |
| Unsorted tissues | 863 |
| Unsorted tumours | 174 |

**S7 Table. Details on non-sperm tissue samples in the GEO analysis.** The corresponding accession numbers are provided in **S8 Table**.
