# Supplementary material for: The DNA methylome of human sperm is distinct from blood with little evidence for tissue-consistent obesity associations
Source: PLoS Genet. 2020 Oct 13;16(10):e1009035. doi: 10.1371/journal.pgen.1009035 (PMC7584170; doi:10.1371/journal.pgen.1009035)
Supplement: S14 Table — DNA methylation age acceleration based on three different estimators was separately regressed onto five weight-related or metabolic traits: BMI, obesity (being in the obese/overweight group), waist circumference, insulin resistance (HOMA-IR) and fasting insulin. None of the 25 associations were significant (P > 0.05 for all tests). (DOCX) [file pgen.1009035.s015.docx]

|  | **Estimator** | Horvath | | PhenoAge | | Jenkins |
| --- | --- | --- | --- | --- | --- | --- |
| **Trait** | **Tissue** | Blood | Sperm | Blood | Sperm | Sperm |
| BMI | P value | 0.33 | 0.84 | 0.08 | 0.72 | 0.20 |
|  | r^2^ | 0.01 | 0.00 | 0.04 | <0.01 | 0.02 |
| Obesity | P value | 0.88 | 0.75 | 0.83 | 0.83 | 0.58 |
|  | r^2^ | <0.01 | 0.01 | 0.00 | <0.01 | 0.02 |
| Waist circumference | P value | 0.39 | 0.88 | 0.08 | 0.81 | 0.14 |
|  | r^2^ | 0.01 | <0.01 | 0.04 | <0.01 | 0.03 |
| HOMA-IR | P value | 0.38 | 0.08 | 0.61 | 0.29 | 0.64 |
|  | r^2^ | 0.01 | 0.05 | <0.01 | 0.02 | <0.01 |
| Fasting insulin | P value | 0.20 | 0.10 | 0.24 | 0.62 | 0.83 |
|  | r^2^ | 0.03 | 0.05 | 0.02 | 0.01 | 0.00 |

**S14 Table. Associations between DNA methylation age acceleration and weight-related or metabolic traits.** DNA methylation age acceleration based on three different estimators was separately regressed onto five weight-related or metabolic traits: BMI, obesity (being in the obese/overweight group), waist circumference, insulin resistance (HOMA-IR) and fasting insulin. None of the 25 associations were significant (P > 0.05 for all tests).
