# Supplementary material for: The DNA methylome of human sperm is distinct from blood with little evidence for tissue-consistent obesity associations
Source: PLoS Genet. 2020 Oct 13;16(10):e1009035. doi: 10.1371/journal.pgen.1009035 (PMC7584170; doi:10.1371/journal.pgen.1009035)
Supplement: S15 Table — Semen sample parameters were measured using the Computer-Assisted Sperm Analysis (CASA)/Sperminator software (Pro-Creative Diagnostics, Staffordshire, UK). V = volume, C = concentration, SD = Standard Deviation, WHO = World Health Organization. Percentage A-D sperm refer to the proportion of spermatozoa in different motility grades where A = most motile and D = least motile. Reference ranges derived from [53]. (DOCX) [file pgen.1009035.s016.docx]

|  | Discovery group | Lean replication group | Obesity/over-weight group | Reference range | P |
| --- | --- | --- | --- | --- | --- |
| Volume (sperm, mL).  Mean (SD) | 2.9 (1.1) | 2.9 (1.4) | 2.6 (1.5) | > 1.5 mL | 0.538 |
| Concentration (sperm, millions). Mean (SD) | 55.4 (37.2) | 47.9 (33.9) | 57.4 (31) | > 15 millions/mL | 0.608 |
| Total count per ejaculate (millions). Mean (SD) | 161 (150.4) | 149 (140.5) | 157 (131.5) | > 39 million | 0.953 |
| Percentage A sperm. Mean (SD) | 14.8 (10.6) | 15.4 (10.6) | 17.4 (10.3) | N/A | 0.610 |
| Percentage B sperm. Mean (SD) | 23.9 (9.5) | 22.1 (9.4) | 20.4 (8.6) | N/A | 0.348 |
| Percentage C sperm. Mean (SD) | 12.1 (3.7) | 11.4 (3.7) | 11.1 (4.3) | N/A | 0.589 |
| Percentage D sperm. Mean (SD) | 49.3 (18.1) | 50.5 (18.7) | 51.1 (18.8) | N/A | 0.926 |
| Average motile speed. Mean (SD) | 18.6 (2.6) | 19.2 (4.4) | 19.4 (2.3) | N/A | 0.603 |

**S15 Table. Semen sample parameters for the discovery and replication groups (the lean replication group and the obesity group).** Semen sample parameters were measured using the Computer-Assisted Sperm Analysis (CASA)/Sperminator software (Pro-Creative Diagnostics, Staffordshire, UK). V = volume, C = concentration, SD = Standard Deviation, WHO = World Health Organization. Percentage A-D sperm refer to the proportion of spermatozoa in different motility grades where A = most motile and D = least motile. Reference ranges derived from [3].
