# Supplementary figures and images for: The DNA methylome of human sperm is distinct from blood with little evidence for tissue-consistent obesity associations
Source: PLoS Genet. 2020 Oct 13;16(10):e1009035. doi: 10.1371/journal.pgen.1009035 (PMC7584170; doi:10.1371/journal.pgen.1009035)

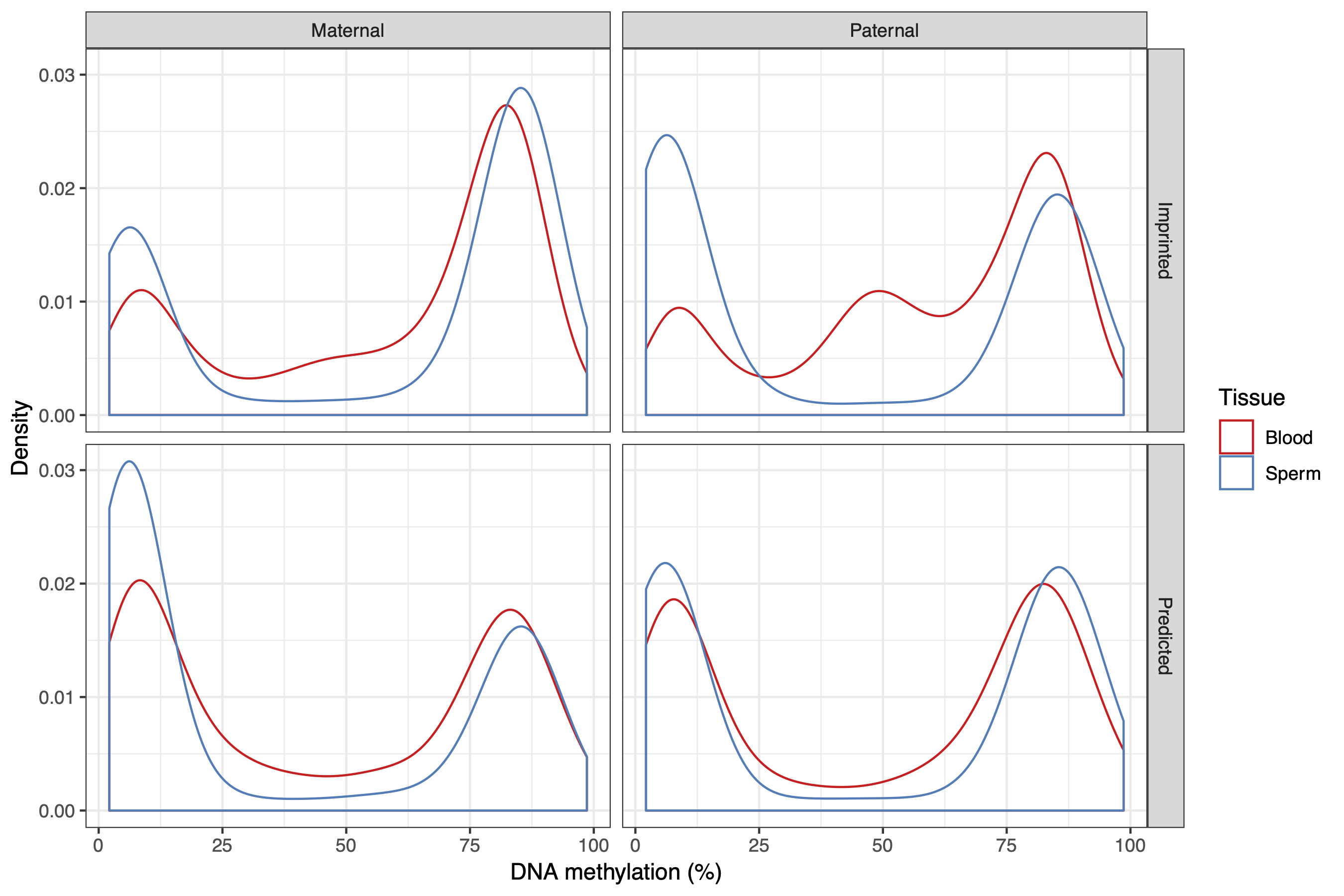

Supplement: S1 Fig — DNA methylation annotated to known imprinted genes (Geneimprint database; http://www.geneimprint.com), showed a characteristic enrichment in sites with DNA methylation around 50% (+/- 10%) in whole blood—particularly, those genes known to be paternally imprinted (P < 1.00 × 10^−50^, Fisher’s exact test), but also for maternally imprinted genes (P = 9.19 × 10^−9^) and a less pronounced enrichment in genes predicted to be imprinted paternally (P = 0.01) or maternally (P = 0.04). No such enrichment was observed in sperm (P > 0.05 for all four tests). (TIFF) [file pgen.1009035.s017.tiff]

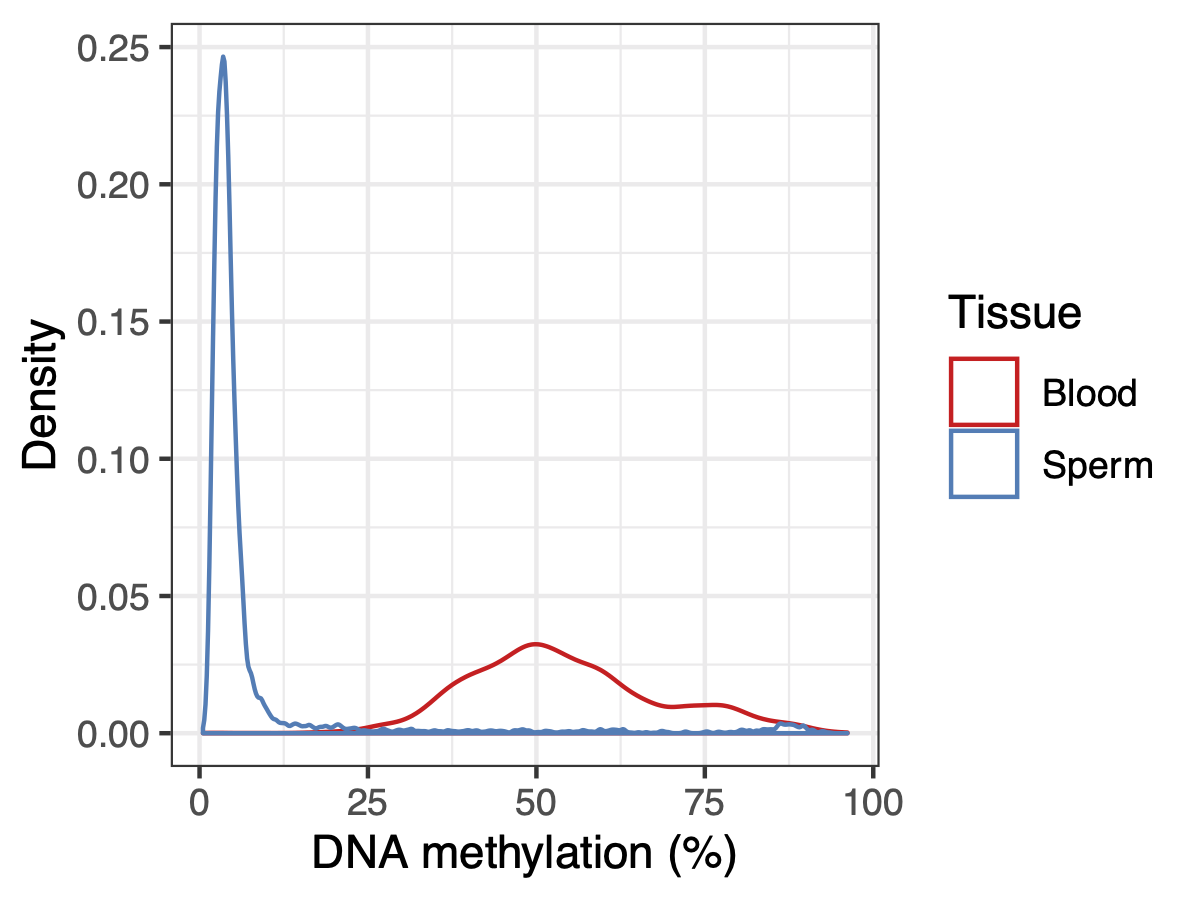

Supplement: S2 Fig — Nearly all of the 169 CpG sites that are located in known imprinting control regions (ICRs) display intermediate DNA methylation levels in blood (57% of sites with median DNA methylation between 40 and 60%; P < 1.00 × 10^−50^, Fisher’s exact test). Simultaneously, they appear to be completely unmethylated in sperm (94% of sites with median DNA methylation < 20%, P < 1.00 × 10^−50^). (TIFF) [file pgen.1009035.s018.tiff]

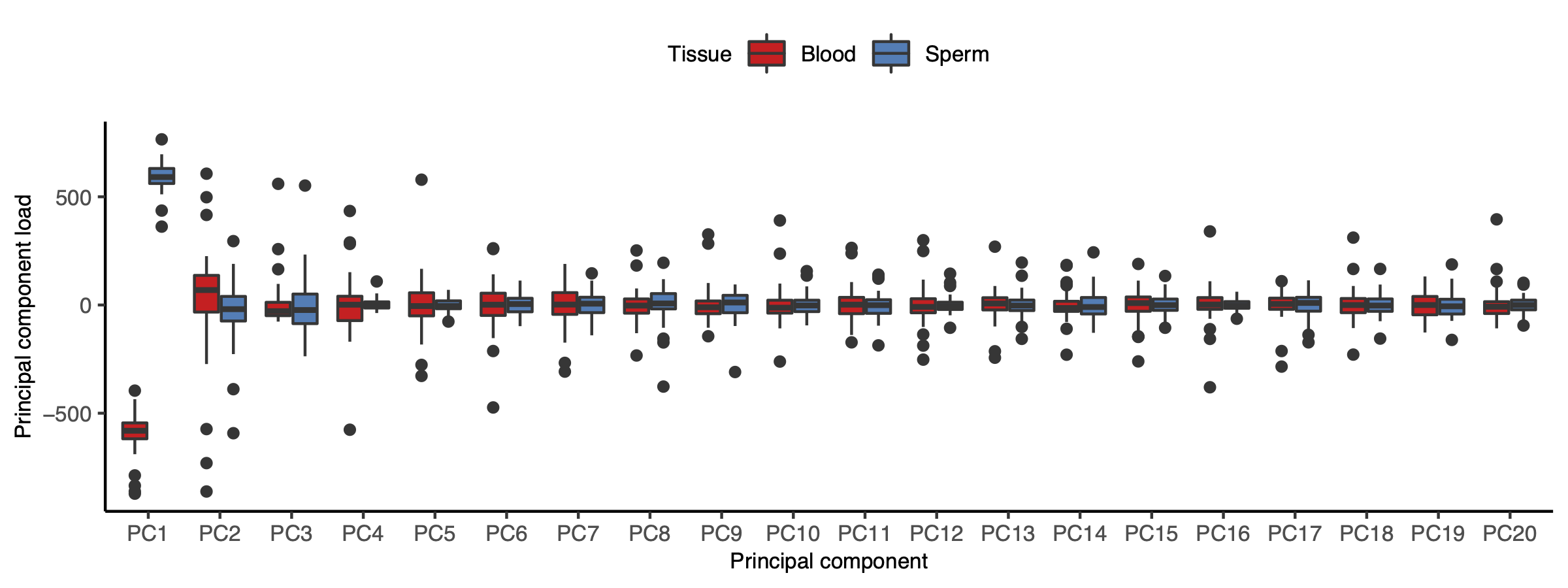

Supplement: S3 Fig — The first PC, which explained 51.41% of the total variance, clearly distinguishes between blood and sperm, making tissue/cell type the single biggest factor contributing to variation in DNA methylation across our samples. (TIFF) [file pgen.1009035.s019.tiff]

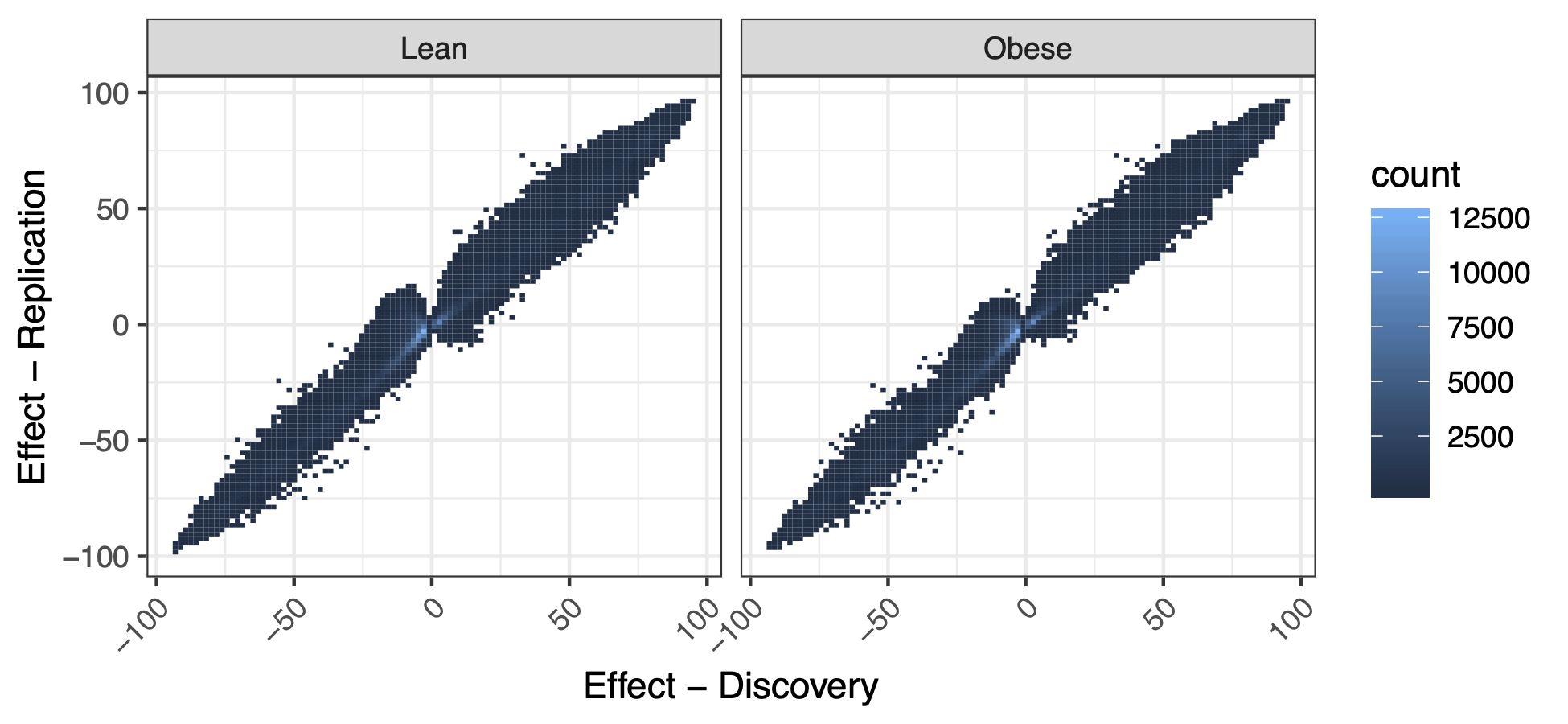

Supplement: S4 Fig — The effect sizes at the 441,764 significant probes from discovery, which were also present in the replication datasets, were highly correlated with those observed in the replication groups (lean group: r = 98%, P < 1.0 × 10^−50^; obese group: r = 0.99, P < 1.0 × 10^−50^). (TIFF) [file pgen.1009035.s020.tiff]

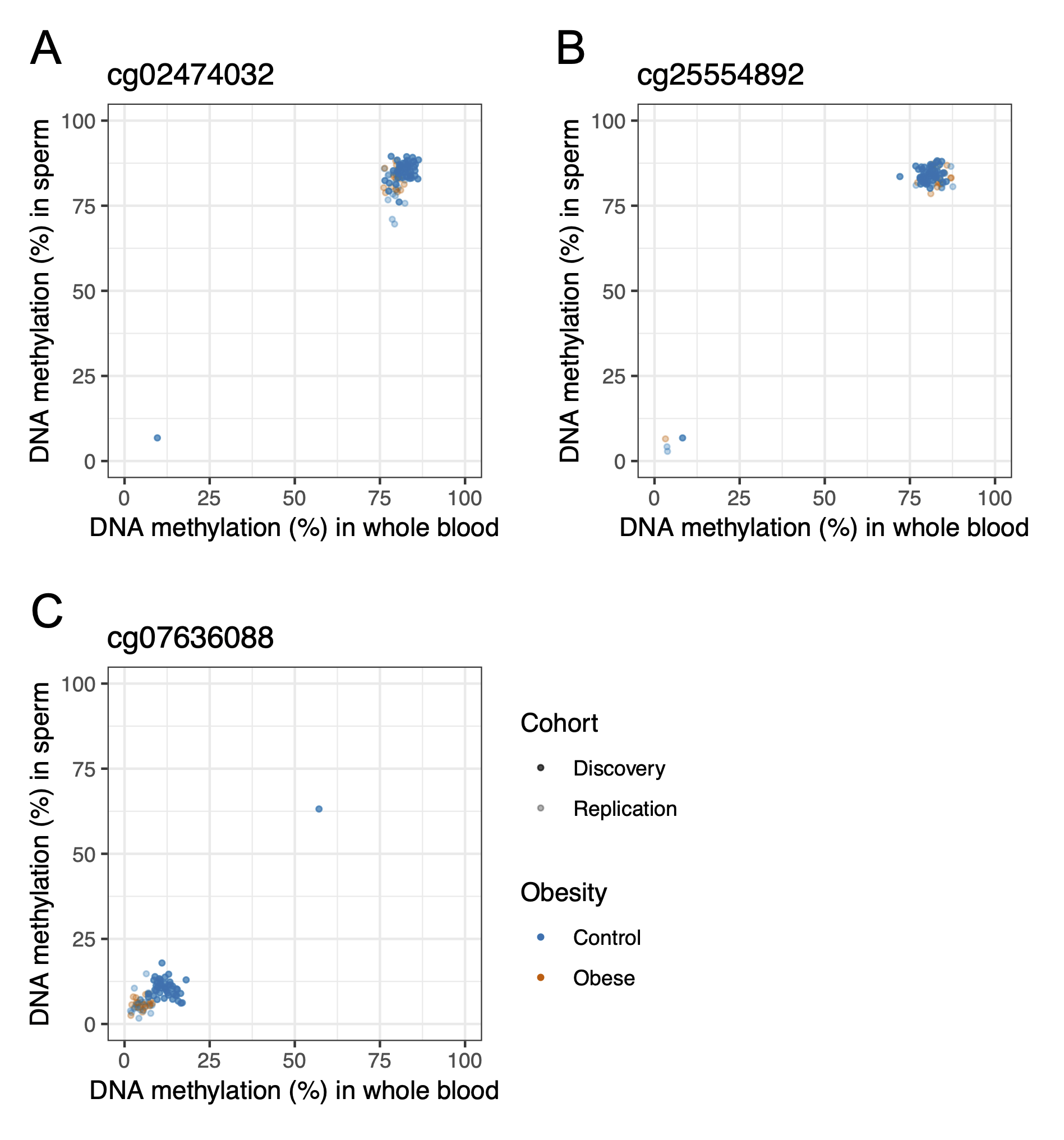

Supplement: S5 Fig — Shown is DNA methylation in whole blood and sperm from the discovery and replication datasets at (A) cg02474032 (chr16:87678659), (B) cg25554892 (chrX:70434406), and (C) cg07636088 (chr13: 31734946). We observed higher measured DNA methylation in the individual outlier at less than 2% of these 365 sites. (TIFF) [file pgen.1009035.s021.tiff]

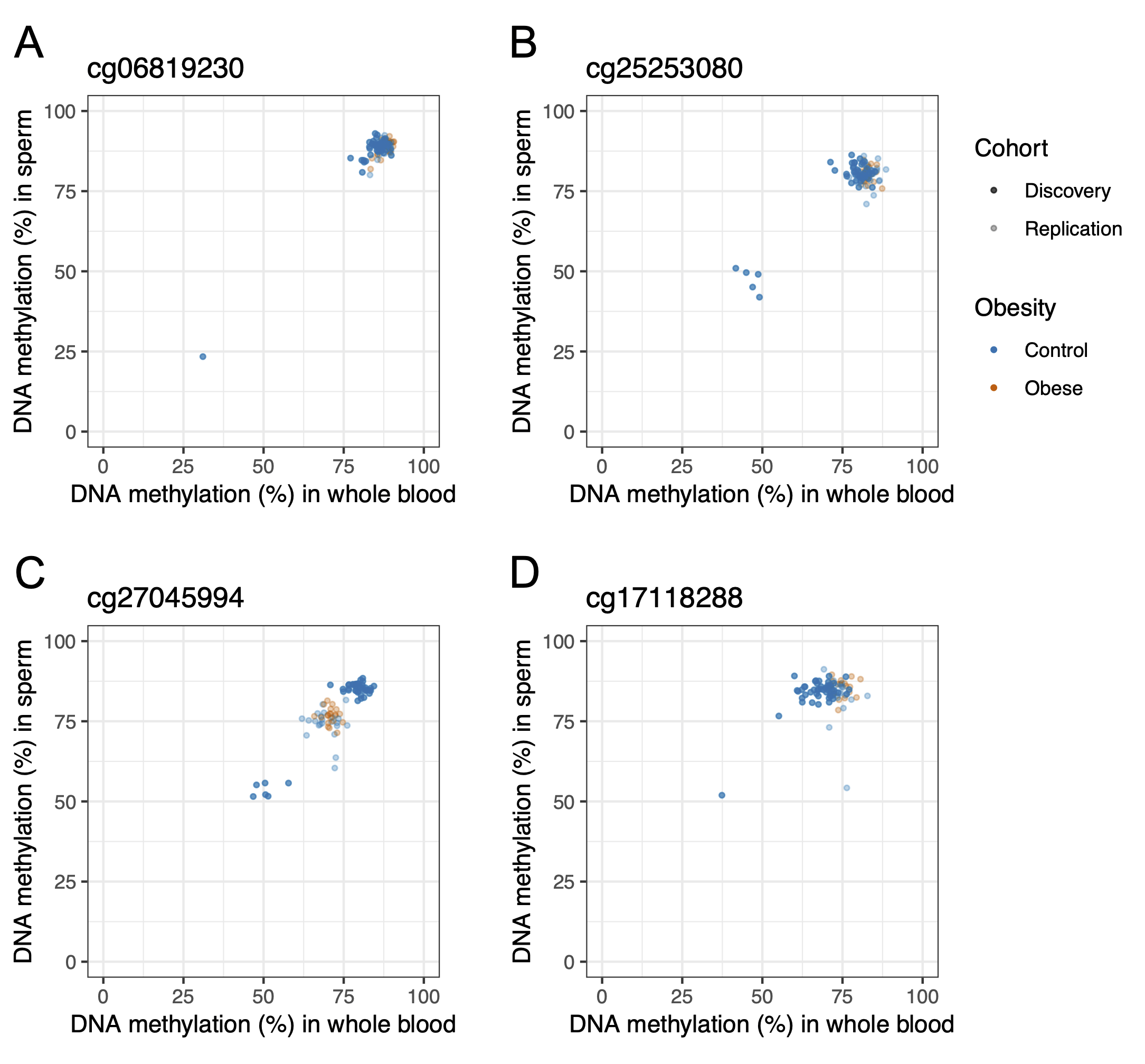

Supplement: S6 Fig — Of the 1,250 correlated probes also present in the replication data 173 (13%) show no evidence of correlation in the replication datasets (r < 0.3 in both datasets) (A) The majority of these sites (127 sites; 76%) were characterized by a single outlier in the discovery data, without any outliers in the replication datasets. One example is found at cg06819230 (chr16:67567158). (B) cg25253080 (chr10:14795564) represents the only incidence where a set of 5 outliers did not replicate in either replication group. (C) The biggest set of outliers which did not replicate contained 6 individuals, with no outliers in the replication data and was found at cg27045994 (chr8:284126). (D) The only trimodal distribution which did not replicate was observed at cg17118288 (chr1:218563763). (TIFF) [file pgen.1009035.s022.tiff]

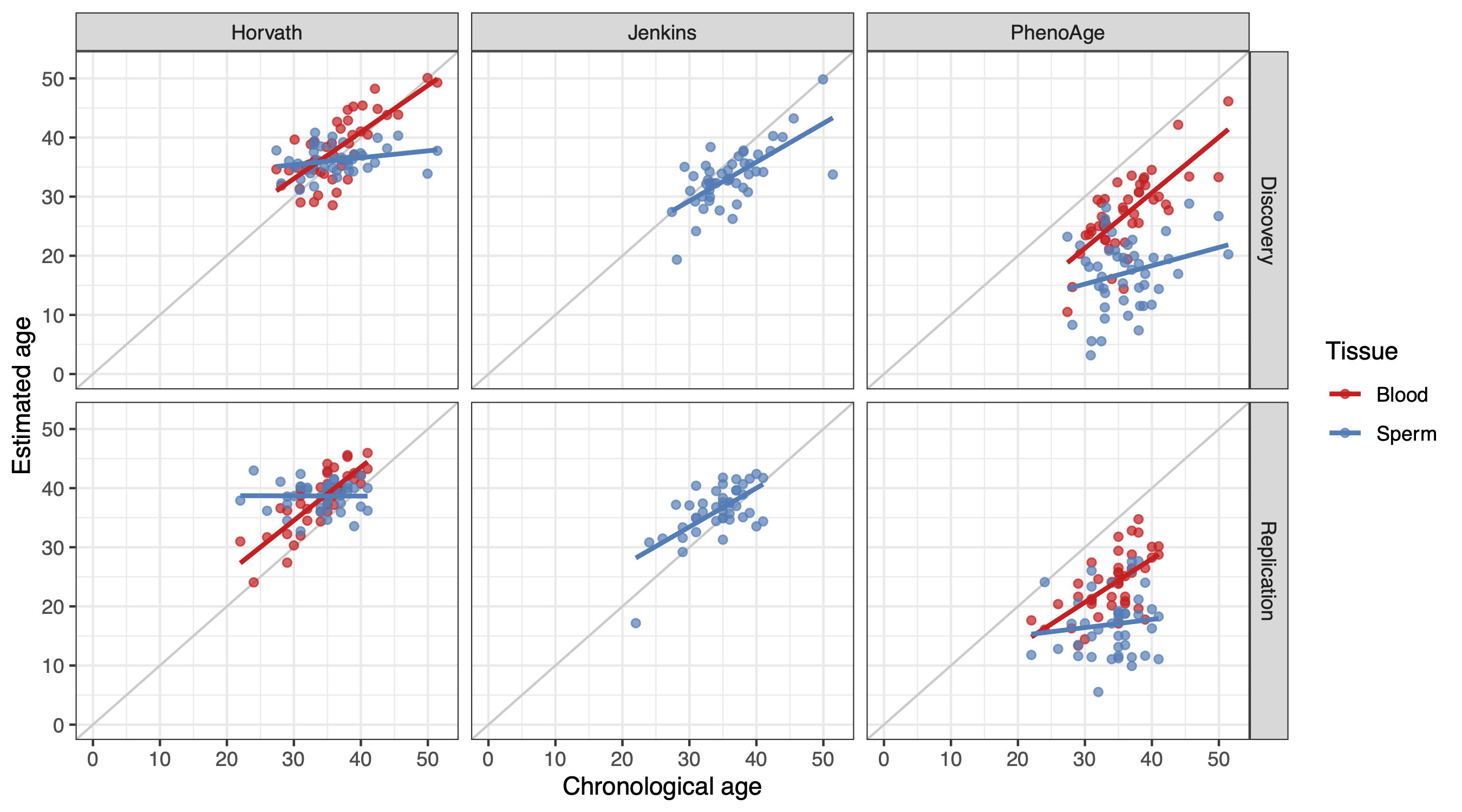

Supplement: S7 Fig — As reported previously, the DNA methylation age predictor by Horvath was significantly correlated with chronological age in whole blood but not in sperm. However, chronological age could be more accurately predicted from DNA methylation in sperm using the predictor more recently developed by Jenkins and colleagues. Like the Horvath methylation age estimator, the PhenoAge estimator showed stronger correlations with chronological age in blood than in sperm. Note that the lean and obese/overweight replication groups were combined into one “replication” group for these analyses. (TIFF) [file pgen.1009035.s023.tiff]

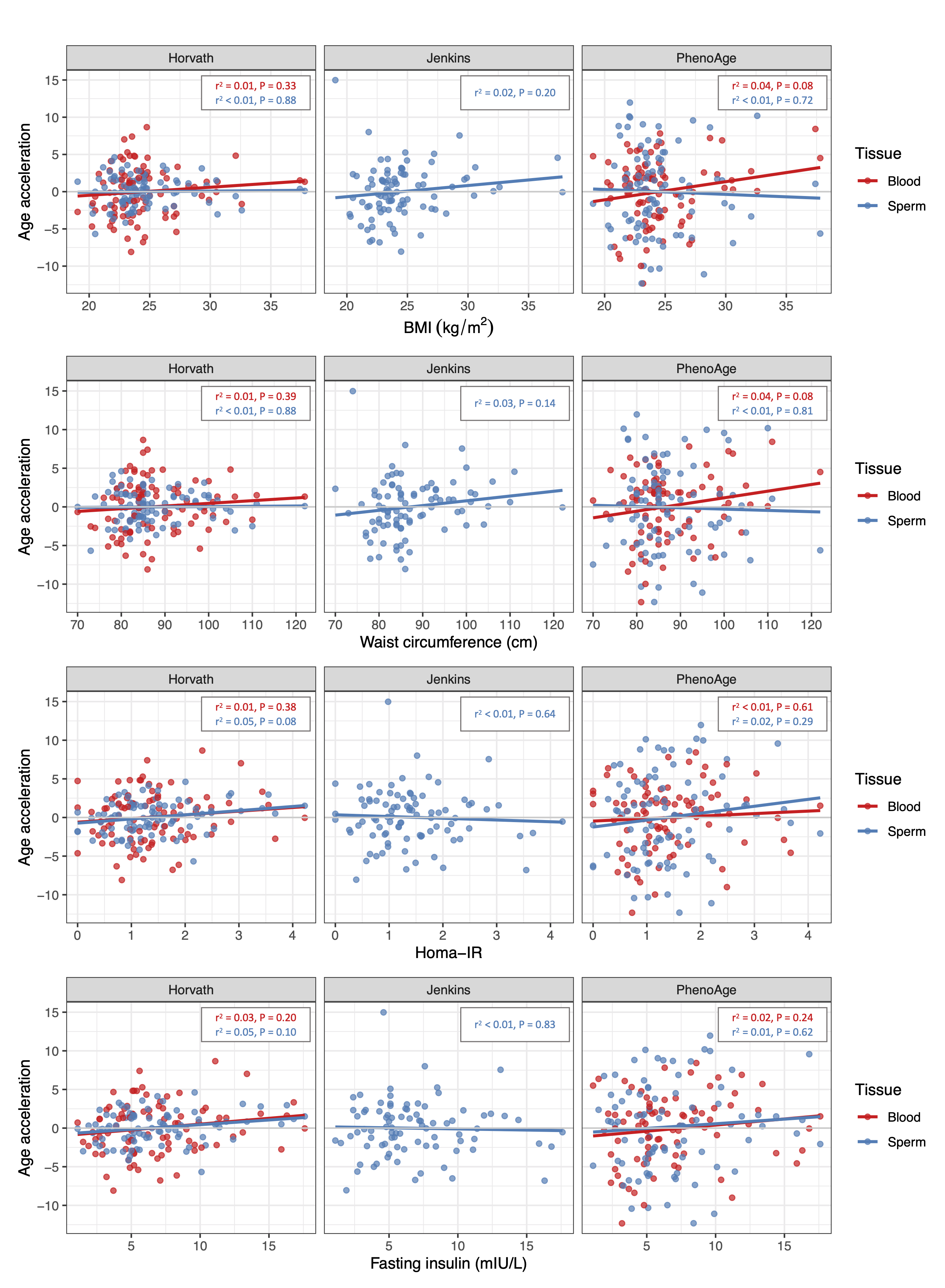

Supplement: S8 Fig — Scatter plots of DNA methylation age acceleration based on three different estimators and four weight-related or metabolic traits (BMI, waist circumference, Homa-IR and fasting insulin) are shown in whole blood and sperm. Linear regressions were performed for each of these 20 comparisons: No significant associations were identified. Note that all discovery and replication groups were combined for this analysis. (TIFF) [file pgen.1009035.s024.tiff]

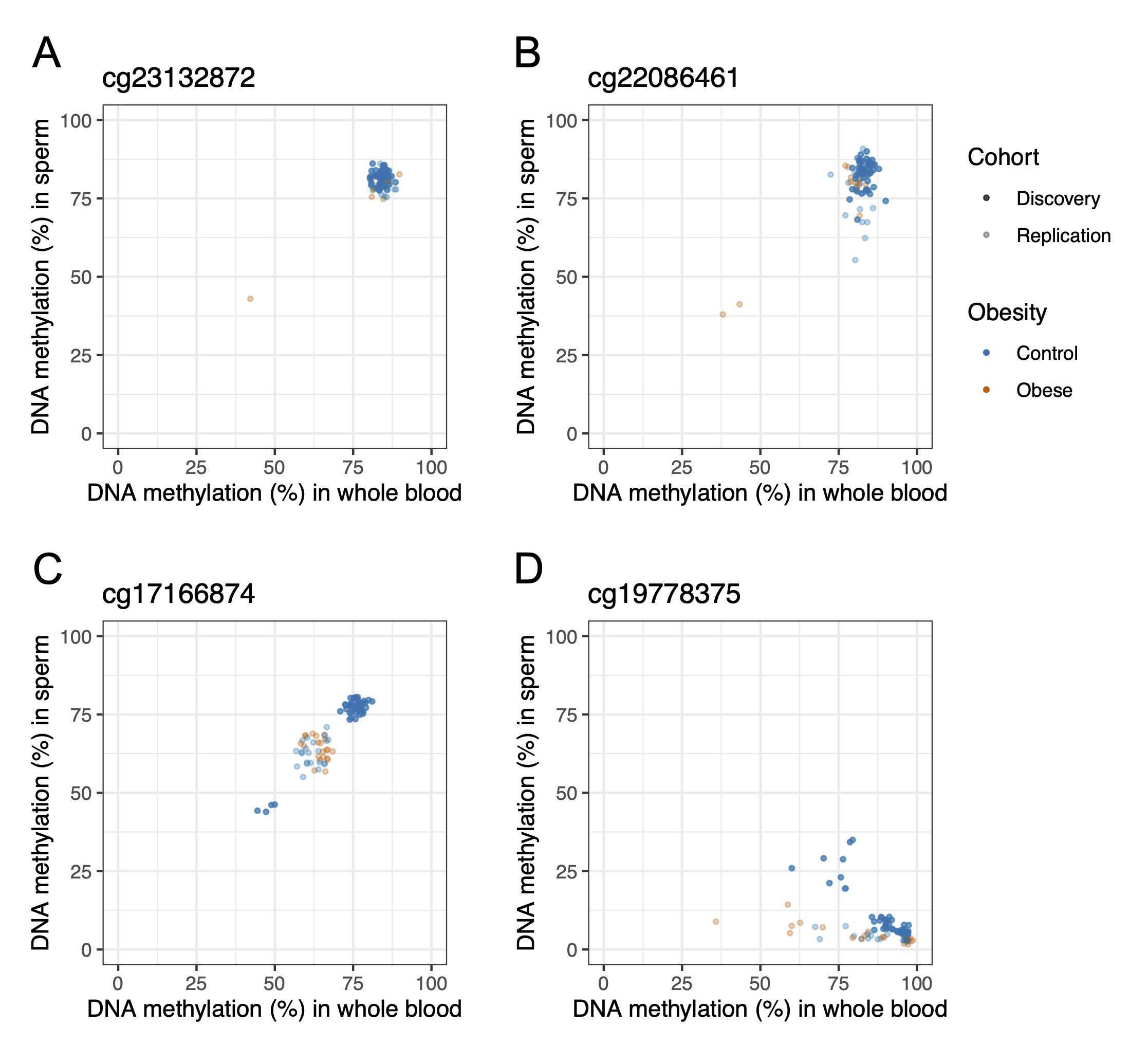

Supplement: S9 Fig — The majority of significant interactions between sperm and blood DNA methylation and obesity were driven by single or very few outliers in the obesity group. (A) At cg23132872 (chr2:191882300), the correlation in obese individuals is driven by a single outlier. (B) At cg22086461 (chr8:77343728) the correlation in obese individuals is driven by two outliers. (C) At cg17166874 (chr7:155381422) the correlation in lean men is driven by four outliers in the discovery data and methylation at this site is also characterized by substantial batch effects. (D) At cg19778375 (chr12:297831) there appears to be a batch effect between the discovery and replication datasets that contributes to an observed correlation in the lean men from the discovery cohort, which is not present in the replication datasets. (TIFF) [file pgen.1009035.s025.tiff]

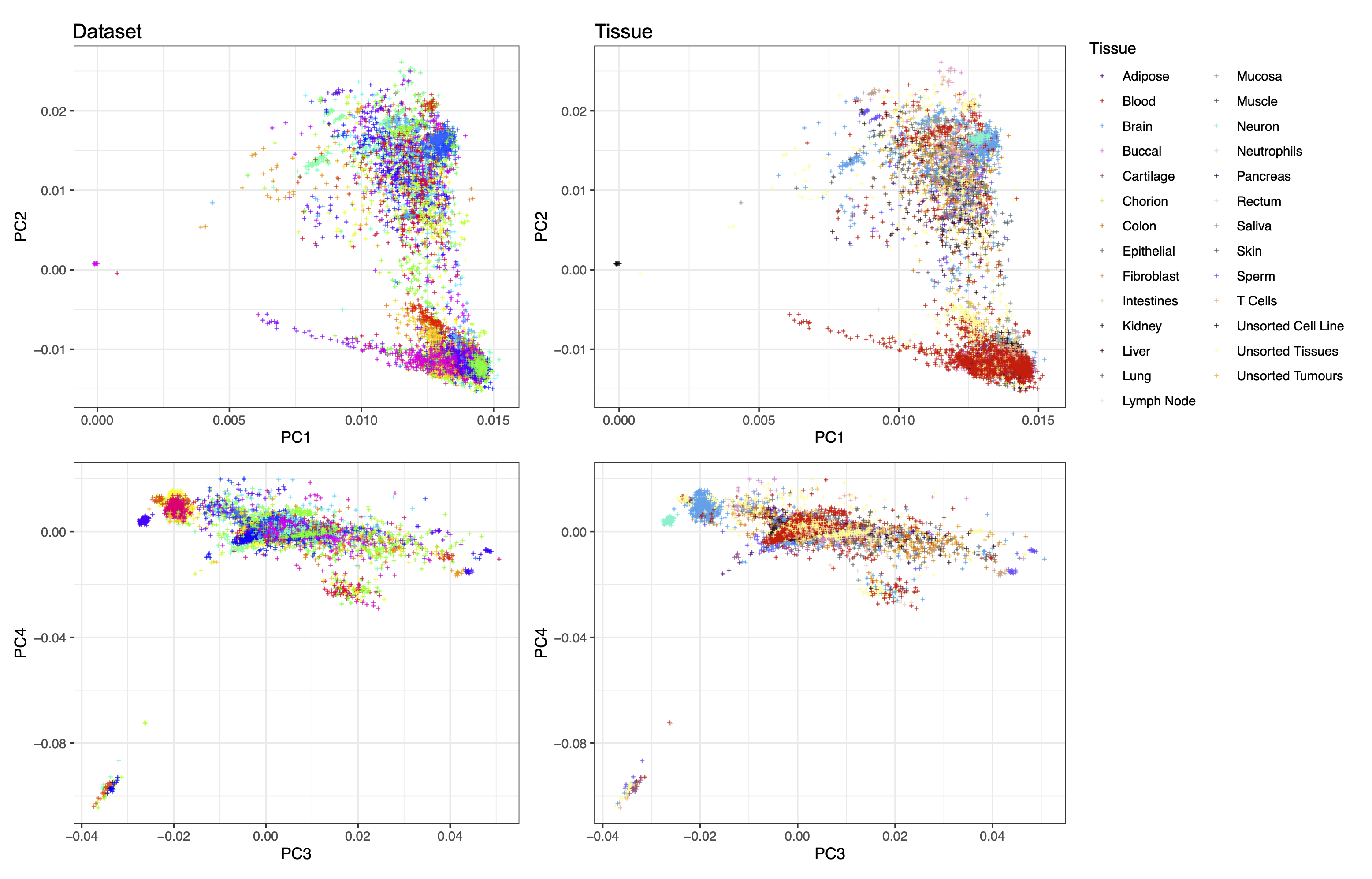

Supplement: S10 Fig — DNA methylation profiles were downloaded from 93 separate datasets on GEO. From a combined PCA, the loads on the first four principal components for each sample are shown here, coloured by dataset of origin and tissue (legend only shown for tissues). While individual datasets often only contain a single tissue of origin and therefore batch and tissue effects may overlap and there are clearly study specific effects, we also see similarities between samples of the same tissue type across datasets. (TIFF) [file pgen.1009035.s026.tiff]
