# Supplementary material for: The DNA methylome of human sperm is distinct from blood with little evidence for tissue-consistent obesity associations
Source: PLoS Genet. 2020 Oct 13;16(10):e1009035. doi: 10.1371/journal.pgen.1009035 (PMC7584170; doi:10.1371/journal.pgen.1009035)
Supplement: Attachment — Submitted filename: PLOS Genetics Response to reviewers.pdf [file pgen.1009035.s027.pdf]

We thank the editors and reviewers for their positive comments. We have made revisions to the manuscript as detailed below.

Reviewer's Responses to Questions

Comments to the Authors:
Please note here if the review is uploaded as an attachment.

Reviewer #1: This study aimed to characterize associations between obesity and DNA methylation of sperm and blood. Furthermore, the study compares 1) the DNA methylation landscape of sperm relative to blood in paired samples and among other non-paired tissues 2) the potential DNA methylation signature of obesity within sperm and blood. Genome-wide DNA methylation of paired blood-sperm samples was profiled with the EPIC array within a limited number of participants. However, the striking differences across tissues enabled the authors to characterize mostly discordant DNA methylation sites and ~1% DNA methylation sites which are concordant. Some limited evidence of an obesity methylation signature across tissues is presented. The study adds to our understanding of tissue specific signatures and the importance of considering genetics in EWAS. However, the limited number of samples and design of the study makes interpretation challenging. There are a several major concerns that need to be addressed below:

We thank Reviewer 1 for their positive comments.

Major:

1. Abstract: The article refers to "effect" of obesity to DNA methylation. It should be revised to "association" as this study is cross-sectional and correlational in nature. Please rephrase any "effects" to associations for consistency.

We agree with the reviewer and have edited the manuscript to replace "effect" with "association" throughout the manuscript, when referring to the association between obesity and DNA methylation in our data.

2. Abstract: "Obesity only nominally influences sperm DNA methylation, making it an unlikely mediator of intergenerational effects of metabolic traits." Given the sample size and design, this study has limited power to make such generalizable conclusions. Please rephrase to state that this was observed in this study only.

We thank the reviewer for this comment and have and have toned down the sentence in question:

"We observed that obesity was only nominally associated with differential DNA methylation in sperm, and therefore suggest that spermatozoal DNA methylation is an unlikely mediator of intergenerational effects of metabolic traits."

3. Author summary: The statement “although more research is needed, obesity in fathers may not affect the health of their children directly.” This is out of the scope of this paper, the paper only tested obesity DNA methylation associations no inferences can be made about many health endpoints which have been shown in rigorous epidemiological studies.

We agree with the reviewer and feel like we may have gone overboard with this sentence in the author summary. We have amended the author summary to specifically only refer to the mediation of intergenerational inheritance of obesity via DNA methylation.

4. Results: The analyses of epigenetic clocks are surprising in the results as this is not mentioned as a hypothesis. Additionally, within the scope of the paper would be to test associations with obesity influencing epigenetic age but the authors don’t do this. I suggest leaving out or further elaborating on obesity and epigenetic aging. Particularly for the sperm clock.

We thank the reviewer for this helpful suggestion. Both reviewer 1 and 2 commented on the DNA methylation age analyses, suggesting further analyses of DNA methylation age acceleration and obesity. We have undertaken new analyses of DNA methylation age acceleration with five weight-related or metabolic traits using three different age estimators (including PhenoAge, as suggested by reviewer 2). None of these analyses found any significant associations. These new analyses are now described and motivated in the ‘Results’ section after the probe-wise association of obesity and DNA methylation, in the ‘Materials and Methods’ section and in S7 Fig, S8 Fig and S13 Table.

5. Results: The trimodal distribution as a criterion of genetic control needs to be further explained. Are the authors suggesting that these measurements reflect a measurement of a SNP? (i.e. AA, AB, BB)? Please male sure to distinguish from meQTLs. As of now it is difficult to know if the authors suggest SNPs at the site/probe or meQTLs.

We fully agree that it is difficult (impossible without additional genotyping data) to distinguish between CpG-SNPs, meQTL effects or even technical measurement effects of SNPs in probes. We have added the following sentence to the ‘Results’ to highlight this:

“Such effects could for example arise from SNPs in the CpG sites themselves (where the methylation value would represent a genotype call rather than methylation measurement), from very strong mQTLs leading to three distinct levels of methylation for the three genotypes at the QTL, or possibly because a SNP in the probe sequence is biasing the measurement of DNA methylation.”

6. Results: Why was BMI chosen to be modeled as categorial, the authors need to elaborate between the modeling choice (continuous BMI vs categorical classification). One would expect to have greater power if modeling BMI as continuous. Additionally, it would be of high interest to see a scatter plot of cg19357369 DNA methylation and BMI as continuous.

We are thankful for this feedback. With the cost constraints of performing DNA methylation analyses using the MethylationEPIC array, which limited the number of samples we were able to analyse, we opted to investigate the extremes of BMI distribution. Thus, we investigated two groups of individuals with clear differences in metabolic health (please see table S4 for further information), as this increased our chances of detecting obesity-associated differential DNA methylation in spermatozoa. We also note that the distribution of BMI in the obese/overweight group is highly uneven with a few outliers in the very high BMI range:

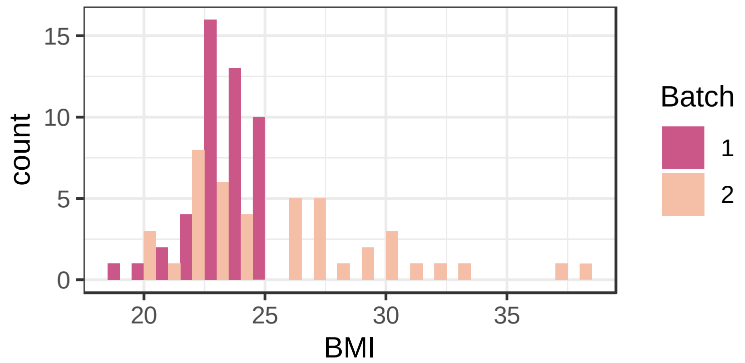

We did not want our analyses to be biased by these outliers and thus opted to for a dichotomous trait. For the interest of reviewer 2 we ran the mixed effects analysis as continuous, which identified a different single significant probe: cg05605371, P = 2.74e-08, effect size = 0.2% and plotted the probe cg19357369 as continuous (P = 1.99e-05, effect size = 0.1%):

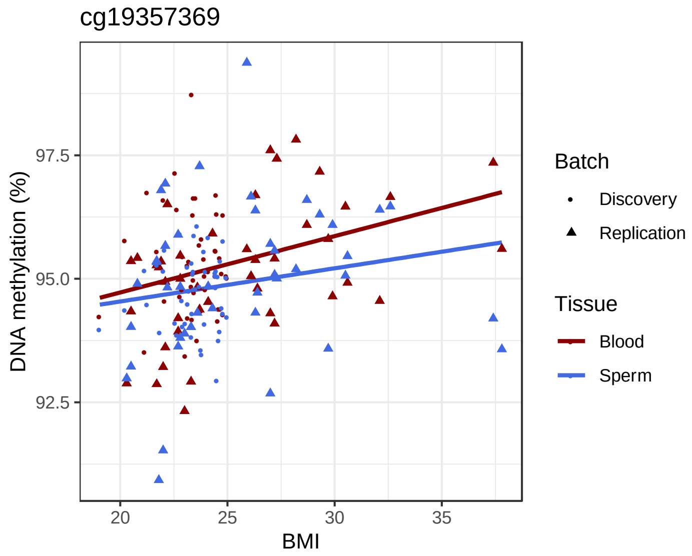

7. In the methods no mention on how and when BMI was measured is presented. Need to elaborate, clinical visits? Self-report? and timing of ascertainment (i.e. during sample collection).

We apologise for not providing enough detail previously and have amended the 'Samples' section under 'Materials and Methods' accordingly, including a detailed description of BMI measurement:

"To determine BMI, participants were weighed wearing only light clothing and their height was measured by a trained researcher during the same research clinic visit as when their blood samples were taken, and within two weeks of providing a sperm sample."

8. Discussion: a previous study reported associations between periconception obesity and child DNA methylation and birthweight (https://jamanetwork.com/journals/jamanetworkopen/fullarticle/2757997). It would be of high interest to do a reverse look-up of the 9 sites reported in that paper and findings in sperm and blood within this study.

We thank the reviewer for this helpful suggestion and pointing out this study to us. We have performed the suggested lookups of DNA methylation at the nine sites and included this in the results section after (P., l.). One probe showed a nominally significant association with obesity in sperm and the mixed effects model, which is promising. But we remain cautious given how weak the association is and the fact that it would not survive correction for the 18 tests conducted.

Minor:
Abstract: please rephrased "Obesity only nominally influences sperm DNA methylation" given that the study was cross-section it would be more accurate to state "Obesity was nominally associated with sperm DNA methylation".

We are thankful for this constructive feedback and have edited the manuscript accordingly.

Reviewer #2: This paper describes a study of DNA methylation in matched blood and sperm samples in relation to obesity (n=22 obese, n=68) not obese). The study conducts a number of interesting and relevant analyses, including: it characterises and compares the distribution of DNA methylation across the genome, and particularly in imprinted regions, in both tissues; it calculates the correlation between chronological age and epigenetic age predicted by DNAm in both tissues; it compares the DNAm profile of sperm and that of somatic tissues using publicly available data; it calculates the correlation between blood and sperm at variable sites; it explores the likelihood of any blood-sperm correlations being driven by SNPs; it studies the association between obesity status and DNAm in blood and sperm in discovery and replication datasets; it explores enrichment for previously-identified BMI-associated CpGs in the blood and sperm EWAS results. The study concludes that the DNA methylation profiles of sperm and blood are highly distinct, and that there is little evidence of DNA methylation covariation between the two tissues, beyond genetic and technical effects.

The paper is well-written, clear and very interesting. It will be a valuable addition to the literature, which so far (perhaps partly attributable to publication bias) suggests paternal obesity and sperm methylation (and offspring methylation) are correlated. Most of these studies have looked at candidate genes and the field is in need of a genome-wide study with robust and comprehensive methods like this one.

I have only minor suggestions for how the manuscript might be improved.

We are very grateful to Reviewer 2 for their positive comments.

Line 87-88 "[paternal factors] have the potential to negatively impact the development and physiology of a man's offspring, presumably via alterations to his spermatozoa." This sentence should be made more specific, or elaborated on to include other possibilities. Although changes to sperm are the most likely mechanisms responsible for direct prenatal effects, there are other possibilities, such as changes to the other constituents of semen and indirect effects via the mother. Of course, postnatal direct effects (e.g. through paternal behaviour) are also likely. For the same reason, I also suggest that in places where the authors state that their findings suggest that DNA methyaltion is an unlikely mechanism for intergenerational effects of obesity in humans (e.g. line 398), the wording be clarified (e.g. SPERM methylation, to distinguish from the possibility of paternal effects on maternal methylation or postnatal offspring methylation).

We fully agree with this comment and have edited the manuscript accordingly. The introduction now includes the following sentence:

"Such associations could be mediated by alterations to the father's spermatozoa (**Fig 1A**), although other possibilities include changes in the composition of seminal fluid or indirect effects on the mother and, importantly, postnatal effects such as paternal behaviour."

We now refer to sperm DNA methylation specifically, when referring to a mechanism for intergenerational effects.

The authors have calculated the epigenetic age of sperm using the Horvath approach, and then calculated the correlation with, I think, chronological age (presumably this is what is meant by "works well"). The rationale for this analysis is not given but should be. Have the authors considered also looking at other epigenetic clocks, including phenoAge, and looking at the correlation between epigenetic age (acceleration) and obesity status?

We thank the reviewer for this helpful suggestion. Both reviewer 1 and 2 commented on the DNA methylation age analyses, suggesting further analyses of DNA methylation age acceleration and obesity. We have undertaken new analyses of DNA methylation age acceleration with five weight-related or metabolic traits using three different age estimators (including PhenoAge, as suggested by reviewer 2). None of these analyses found any significant associations. These new analyses are now described and motivated in the results section after

the probe-wise association of obesity and DNA methylation, in the materials & methods section and in S7 Fig, S8 Fig and S13 Table.

The study compares DNAm in sperm (90 samples collected for this study, 281 publicly-available in GEO) to that of 5,917 somatic tissues from male participants from several other studies in GEO. They find that nearly 30% of the tested CpGs are differentially methylated after Bonferroni correction. They then carry out some functional enrichment analyses on the differentially methylated CpGs and find enrichment for gene transcription, neurological traits and sensory traits. They explain that the enrichment for neurological traits is possibly explained by the large proportion of brain and neuronal samples amongst the somatic tissues used. I would like to see how all samples cluster, for example in PCA plots, to be reassured that the major source of variation between sperm and somatic tissues is in fact tissue type and not technical variation introduced by the fact that the dfferent tissues will have been analysed by different labs using potentially different methods.

We have undertaken PCA of the GEO data and have added PCA plots for the first four principal component loads, coloured by dataset of origin (93 separate GEO datasets) and tissue, respectively. Overall, there seems to be little systematic variance across datasets. While individual datasets often only contain a single tissue of origin and therefore batch and tissue effects may overlap and there are clearly study specific effects, we also see similarities between samples of the same tissue type across datasets. A separate PCA including our own EPIC data showed platform (450K vs EPIC) to be a major contributor to overall variance. We therefore repeated the GEO sperm-specific DNA methylation analysis without our own EPIC samples and confirmed that effects remained the same:

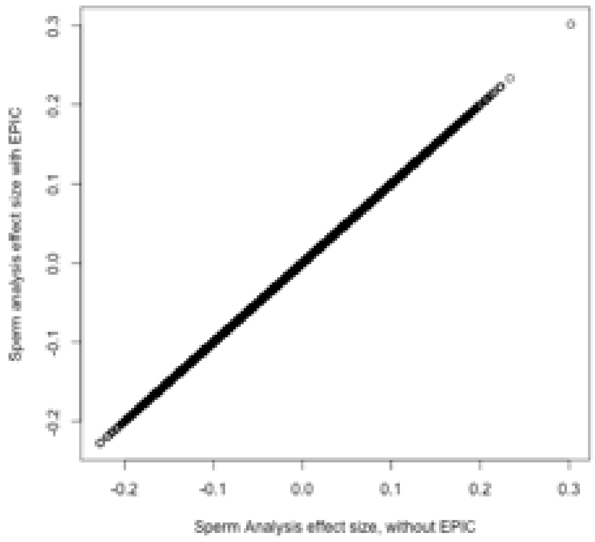

The authors may want to consider a recent preprint that supports the findings of their study: https://www.medrxiv.org/content/10.1101/2020.03.10.20020099v1

We are grateful to Reviewer 2 for alerting us to this interesting and well-written preprint and have made reference to it in the 'Discussion'.

Reviewer #3: Plos Genetics

6/9/2020

DNA methylation covariation in whole blood and sperm: implications for studies of intergeneration epigenetic effects

Summary
Tissue specific epigenetic analyses are important and male semen samples are critically understudied. Authors conduct a tissue specific analysis of blood versus semen DNA methylation in adult males, first with discovery in 47 lean participants and then with replication testing in 21 lean participants. Authors specifically isolated active spermatozoa cell types in the semen samples and they adjusted for cell type proportions in the blood analysis. Widespread and large magnitude epigenetic differences were observed between these two tissues. Authors also conduct an analysis of the association between overweight or obesity status in 22 males versus lean status in both tissues. This weight analysis is likely underpowered and should be considered exploratory. In general, this manuscript is clear, the results are well quantitated, and the tissue specific research question is of high scientific value.

We thank Reviewer 3 for their positive comments.

Critique

• **Authors refer to study samples as cohorts, which implies longitudinal participant follow up. However, this is cross-sectional study. To avoid confusion, these should be described as samples.**

We agree that the term 'cohort' might be taken to imply longitudinal follow-up. We have changed the wording from 'cohort' to 'group' throughout the manuscript and Supplementary Material.

• **Authors use causal language at several points during the manuscript, which overstates the findings based on this study design. For example, in line 53 "We found that there was almost no effect of obesity on sperm". Authors should be very careful to refer to these results as associations rather than causal effects.**

We fully agree with these and similar comments made by reviewer 1. We have changed this specific sentence to

"We found that there was almost no association between obesity and DNA methylation in sperm."
Throughout the manuscript we replaced the term "effect" with "association" when referring to the association between obesity and sperm DNA methylation.

• Epidemiology cross-sectional STROBE reporting guidelines require the study design (cross-sectional study) to be in the title and abstract.

We would like to thank the reviewer for highlighting these guidelines to us. As recommended by the STROBE checklist for cross-sectional studies (https://www.equator-network.org/wp-content/uploads/2015/10/STROBE_checklist_v4_cross-sectional.pdf) the study's design should be indicated by a commonly used term in the title or abstract. We have now included it clearly in the abstract. We have refrained from also including it in the title, because we felt the title was very lengthy already and required some rewording and shortening (see response to following comment). We hope this satisfies the reviewer's request.

• Authors frequently use the terms inheritance and intergenerational effects, however this is a cross-sectional study conducted in a single generation. Generational speculation should be restricted to the discussion and used much more cautiously. From the current title and phrasing throughout, this paper makes promises that are outside the bounds of the available data.

Thank you for this helpful comment. We have edited the manuscript to be more cautious in this regard as highlighted in the text. We have also changed the title of the manuscript to: "**The DNA methylome of human sperm is distinct from blood with little evidence for tissue-consistent obesity associations**", highlighting the tissue differences and lack of tissue-consistency in the obesity association, while steering clear of obesity

• The obese sample is misnamed. In fact, the mean BMI among this group is below 29, which reflects that this sample is overweight or obese.

Thank you for this constructive feedback and we agree that we should have been more modest in our definitions of participant groups. We have now included BMI cut-offs for the participant groups at the first mention of these in the manuscript (also copied below for clarity) to make it clearer to the reader that the obesity group also comprises a proportion of overweight males. We have also clarified that the obesity group includes overweight males in Figure A.

"In this study, we analysed genome-wide DNA methylation using the Infinium MethylationEPIC array in matched samples of human blood and sperm from lean (n = 68; BMI <25kg/m2) and overweight/obese (n = 22; BMI >26kg/m2; 'the obesity group')."

We furthermore added this paragraph to the 'Materials and Methods' section, highlighting the clear metabolic differences between the two groups:

"Two groups of participants were included; lean (BMI <25kg/m^2) and overweight/obese (BMI >26kg/m^2). Phenotypic information about participants is detailed in **S4 Table**, which shows clear differences in metabolic variables between these groups. To determine BMI, participants were weighed wearing only light clothing and their height was measured by a trained researcher during the same research clinic visit as when their blood samples were taken, and within two weeks of providing a sperm sample."

While we still keep the term "obesity group" for plots and repeated references to the group, rather than the lengthier "overweight/obesity group", we trust that the repeated definition of the BMI cut-off and reiteration that this is an overweight/obesity group throughout the manuscript will make this clear to the reader.

• **Recommend adding blood to Figure 1 as well for comparison.**

We would like to thank the reviewer for this suggestion. We have updated the figure accordingly and feel it has improved the clarity of the figure and overview of our study.

• **Authors are adequately powered for the tissue comparisons where effect sizes are expected to be large. In the overweight/obesity versus lean analysis, effect sizes are expected to be much smaller (can estimate from the CHARGE paper). The current study is likely vastly underpowered for this trait analysis. Authors should be more cautious in the interpretations that traits (such as BMI) are not associated with DNAm across these tissues. Perhaps a trait with larger expected effect sizes (such as smoking) with DNAm would be more appropriate benchmark?**

Thank you for this feedback and we agree that smoking (as well as ageing) would be an interesting paternal trait to investigate in future unbiased studies of spermatozoal DNA methylation. We have therefore included a sentence in the 'Discussion' suggesting this should be a priority in future research. In our current study, all participants were non-smokers (we have clarified this is the 'Samples' section under 'Materials and Methods'). We now also refer to a recent systematic review of sperm DNA methylation studies in the 'Discussion' highlighting the scarcity of consistent findings across studies of paternal traits and environmental toxicants.

We agree that our group of overweight/obese participants is small for an EWAS analysis, and that the results of the EWAS in our sample, taken by themselves, should be interpreted as suggestive and exploratory. We believe that we have communicated this as such in the manuscript. What makes us more confident with regards to the lack of a tissue-general association between DNA methylation and BMI is the analysis we undertook using the summary statistics from Wahl and colleagues. This very large study of blood DNA methylation found 184 sites significantly associated with BMI. The fact that we see a significantly enrichment of lower ranked P values and significant correlation of effect sizes with our blood DNA methylation analysis across these sites shows that we have the power to detect enrichments from this much larger study in our sample. Because these enrichments are only seen in our blood and not sperm data, we conclude that if there are sperm DNA methylation profiles associated with BMI

or obesity, they are not the same as those previously identified in blood; and hence there does not appear to be a consistent cross-tissue DNA methylation signature.

• **Probes with multimodal DNAm distributions can be identified agnostically from the data using gaphunter as implemented in the minfi package (PMID: 27980682). This is flexible by sample size and would allow the authors to have criteria beyond visual inspection for flagging these probes.**

We thank the reviewer for highlighting this useful method to us. We have now additionally included the gaphunter method in the 'Results' and 'Materials and Methods' section and found that results based on both methods are largely consistent. Because the gaphunter method only works on one dimensional data (DNA methylation from each of the tissues individually), we still also include our two-dimensional kmeans method. We would like to point out that because gaphunter uses fixed DNA methylation distances (5% by default), its clustering will be affected by the sample size and spread of data. Additionally, as described in the 'Materials and Methods', "visual inspection" was only used for the ~50 probes which performed poorly in the kmeans algorithm, indicated by a between-cluster to within-cluster variance ratio < 2 (and not any random visual criteria). The resulting reclassifications were mostly affected by outliers or probes having more than three clusters in two dimensions (across blood and sperm) and had been misclassified because we had only tested for uni-, bi- and trimodal clusters.

• **Why were the samples preprocessed separately? It is confusing in the results when the total number of probes tested jumps around from 704k to 692k.**

The samples of the discovery and replication groups were processed separately because they are from two completely different batches, recruited and processed at very different timepoints and by different people. We apologize for the confusion caused by the varying numbers of probes tested but we decided that this was the most appropriate way to analyse the data, maximise the power (by including all probes passing QC in each dataset) and communicate details about the analyses undertaken in the manuscript transparently (hence the repeated restating of total numbers of probes tested).

• **Authors refer to the magnitude of association observed using beta. For example, "beta=0.2". Since Illumina refers to beta values of methylation, there can often be confusion between beta values and beta coefficients. Recommend that the authors more fully interpret these coefficients in the text, for example, Overweight or obese participants had 20% higher DNA methylation than lean participants at X site.**

We had originally changed these from DNA methylation percentages to beta values to avoid talking about percentages of percentages in parts of the manuscript (e.g. "57% of sites with 40-60% DNA methylation"). We agree that the difference between beta coefficients and beta values is also (and possibly more) confusing and have now changed everything back to DNA methylation as percentage throughout the manuscript for consistency.

• Can the authors please confirm that IDAT files are available at the GEO link and not just raw and processed methylation values?

Per GEO specification (https://www.ncbi.nlm.nih.gov/geo/info/geo_illu.html) for Illumina array data "IDAT and associated files or a Matrix worksheet containing non-normalized data" are required. For DNA methylation arrays, this raw matrix contains detection P values as well as methylated and unmethylated signal intensities for each probe on the EPIC array. This is the format in which we submitted our data to GEO. From this filtering based on detection P values and calculation of beta values should be straightforward. If the reviewer still feels that IDAT files would be essential beyond this raw matrix format suggested by GEO, we could contact the GEO administrators to amend our submission.

• Is code available to conduct the analyses?

We thank the reviewer for pointing this out, because we had meant to make the code publicly available anyway. We have now made all custom R scripts available in a public GitHub repository: https://github.com/SarahMarzi/BloodSperm_DNAMethylation and added a corresponding note to the data availability statement:

"Analysis code is publicly available on GitHub: https://github.com/SarahMarzi/BloodSperm_DNAMethylation."
